# A new two-step road extraction method in high resolution remote sensing images

**DOI:** 10.1371/journal.pone.0305933

**Published:** 2024-07-18

**Authors:** Wei Lu, Xiaoying Shi, Zhiping Lu

**Affiliations:** Jiangsu Vocational College of Information Technology, Wuxi, China; National University of Sciences and Technology NUST, PAKISTAN

## Abstract

High-resolution remote sensing technology is an efficient and low-cost space-to-earth observation strategy, which can carry out simultaneous monitoring of large-scale areas. It has incomparable advantages over ground monitoring solutions. Traditional road extraction methods are mainly based on image processing techniques. These methods usually only use one or a few features of images, which is difficult to fully deal with the real situation of roads. This work proposes a two-steps network for the road extraction. First, we optimize a pix2pix model for image translation to obtain the required map style image. Images output by the optimized model is full of road features and can relief the occlusion issues. It can intuitively reflect information such as the position, shape and size of the road. After that, we propose a new FusionLinkNet model, which has a strong stability in the road information by fusing the DenseNet, ResNet and LinkNet. Experiments show that our accuracy and learning rate have been improved. The MIOU (Mean Intersection Over Union) value of the proposed model in road extraction is over 80% in both DeepGlobe and Massachusetts road dataset. The figures are available from https://github.com/jsit-luwei/training-dataset.

## Introduction

Recently, high-resolution remote sensing data have the characteristics of wide image coverage, which benefits the scene understanding by the computer vision technology [1]. For example, in areas of urban planning, traffic construction, and environmental monitoring, the accuracy and completeness of road information is crucial to the smart city. The automatic extraction of road information can also reduce labor costs and time consumption, which provides support for research in various fields. Fast and accurate acquisition of information such as road network and lane width is helpful for the design and optimization of traffic roads. In urban planning, automatic extraction of road information can provide a more comprehensive understanding of environmental changes around roads. Therefore, how to use high-resolution remote sensing technology is significant for road information extraction. Traditional road extraction methods are mainly based on image processing, such as edge detection and segmentation. These methods usually use one or a few features of images, which is difficult to fully capture the real situation of the road. In recent years, deep learning technology has been widely used in information extraction. Road extraction methods based on convolutional neural networks (CNN) become one of the typical methods. This kind of methods automatically extract road features from high-resolution remote sensing images by constructing multi-layer networks. They combine subsequent processing to obtain accurate road information using an end-to-end strategy. Contributions of this work focus on the network design and accuracy improvement, which can be summarized as follows.

(1) We optimize a pix2pix model to input data for training rather than the original rough occluded road images. Experiments show that this model is more significant in the case of detecting occluded regions.

(2) We construct a FusionLinkNet model for improving the training and testing performance by optimizing net structure to ensure the convergence.

Experiments show that the proposed road extraction algorithm has a strong stability in the road information extraction. Compared with the same type of different architectures, the accuracy and learning rate have been improved significantly.

The content of this work is as follows. Section 1 introduces the background and research significance of high-resolution remote sensing images in road extraction. Section 2 illustrates the relevant knowledge and classic models of this study. Section 3 shows an analysis of the proposed model. The model structure diagram is drawn in detail, showing the scale of each layer of neural networks. Section 4 presents the relevant data sets, and the qualitative and quantitative analysis. Section 5 provides a summary and outlook, and we analyze the innovation and shortcomings of the proposed research.

## Related works

Generally speaking, object extraction can be divided into semi-automatic and fully automated methods [2]. Semi-automatic road extraction belongs to human-computer interaction. In the fully automatic extraction method, the computer automatically and accurately locates the position of the road by recognizing and understanding image features. At present, the automatic extraction method is difficult to be fully realized. Therefore, we divide the road extraction methods into the traditional methods and the deep learning methods [3].

In the traditional methods, there are three classicial algorithms, namely the template matching, knowledge-driven and object-oriented. The template matching method is to comprehensively utilize the geometric, radiation and topological features of images. Usually, there are three steps. (1) Template design. This can be manually set using certain rules. (2) Measure analysis. When given a template, we search for the extreme values of the region within a specified area through a measurement function. (3) Dynamically update. This is based on dynamically updating the road position. The commonly used templates in the design process include regular templates and variable templates. The difference is that the templates in the regular templates can be described by regular graphics such as circles and rectangles [4]. The shape and size of the variable template depends on the imaging process and the characteristics of the target object. Since the cross-sectional image of the road is easily affected by dark and bright spots, such as road vehicles and shadows, the researchers constructed a rectangular template. However, the template cannot be well applied to curves with large curvature, so Fu et al. [5] proposed a circular template, which takes the middle of the road as the center and the width of the road as the template diameter. This tempalte is better adapted to the curve.

However, as the image resolution increases, vehicle occlusion and shadow interference from houses and trees on both sides of the road become the main factors affecting the extraction results. Lin et al. [6] proposed a T-shaped template, which uses a bisect template and a rectangular template to complement each other in matching and tracking, which can better solve the above-mentioned problems. However, this template is sensitive to changes in road radiation values and requires a lot of manual positioning. In order to realize the semi-automatic, typical methods include dynamic contour model and level set model [7]. The dynamic contour model is also known as the Snake model, which expresses the radiation and geometric characteristics of road images with energy functions, and obtains road contours by finding the minimum value of the function. The level set model can effectively deal with the geometric topological changes of closed curves. Compared with regular templates, variable templates are more suitable for road extraction where geometric and radial features change frequently.

Knowledge-driven methods are combined with road-related knowledge, and focuse on establishing a hypothesis testing model between knowledge and image processing results. According to the relationship between knowledge and road features, knowledge can be divided into geometric knowledge, contextual knowledge and other knowledge. The most commonly used knowledge in the extraction method is based on road geometry knowledge, including parallel edges [8] and path morphology [9]. The road has double edges, so it is a kind of parallel edge, and we can use the method of edge extraction to obtain roads. This kind of methods is relatively simple and easy to implement, but the extraction effect is not well for blurred and occluded road edges.

Path morphology has obvious advantages in extracting narrow and long structures in images. It relies more on the results of binary image segmentation, which leads to occlusion and blurring of road images and still affects the extraction effect. The contextual knowledge of the road includes vegetation, buildings, vehicles, indicator lines, etc. Road context knowledge can be used as a basis for supplementary road machine interpretation, but the extraction of vehicle and zebra crossing information belongs to the problem of object recognition, which will increase the complexity of the algorithm. Solving this problem can address the problem of road breakage caused by obstructions. There are many kinds of road knowledge, such as GPS data, vector data, etc. Hence, multi-source remote sensing data fusion technology [10] can realize the analysis of different angles, heights, and scales of target objects. This kind of solution can combine the advantages of different data to provide assistance for road extraction. Although vector data such as geographic information systems and topographic maps lag behind remote sensing images, they are superior in high precision and reliability. Effective information in vector data can partially replace manual intervention when obtaining certain initial road information. Using the advantage of massive information is also a way to improve the automation of road extraction.

The object-oriented method [11] regards the road as the basic unit of image analysis, and this analysis method gathers and utilizes the “homogeneous” elements that show the characteristics of the target. The steps of the object-oriented road extraction method include pre-processing, image segmentation, image classification, and post-processing. In the step of image segmentation, simple threshold segmentation is commonly used [12]. The basic idea of threshold segmentation method is to select a reasonable threshold according to the texture difference between road and non-road areas. Then, it determines the region ownership of each pixel points in the image. The noise problem can be better solved by image segmentation. In the actual image, the geometric radiation characteristics of many ground object information are similar, so image classification methods are needed to further process the segmented image. Support vector machine (SVM) [13] is one of the effective methods to solve the classification problem. It maps the data non linearly to the high-dimensional feature space through the kernel function, constructs the optimal classification hyperplane with low dimension. According to the structural risk minimization principle, it finds the function with the minimum expected risk as the discriminant function. Classification cannot solve the problems of blurred and broken road boundaries caused by the initial segmentation unit, which requires post-processing to obtain accurate results, such as mathematical morphology open operation and image difference. The object-oriented method can make full use of the geometric structure characteristics and spectral characteristics of the road. It introduces the spatial characteristics and effectively utilizes the semantic relationship between image objects.

The motivation of deep learning methods is to imitate a neural network of human brain for analysis and learning. It is often used in complex fields, such as target detection, image recognition, semantic segmentation and natural language processing. The task of road extraction based on remote sensing images belongs to the problem of image interpretation. Deep learning methods can provide a new way for the further development of semantic direction interpretation. Karen and Andrew [14] improve the degree of automation greatly, which exceeded the traditional road extraction method. The VGG model increases the depth of the network and reduces the scale of the convolution kernel, which verifies that the small-sized convolution kernel has great advantages. The fully convolutional neural network (FCN) [15] became the most popular model in image semantic segmentation. It replaced the fully connected layer with a convolutional layer, which not only better integrated the full image information but also improved the segmentation efficiency. The DenseNet network structure proposed by Xu et al. [16] builds a road extraction module from local recognition to global perception. Although it can improve the recognition accuracy of unoccluded roads, it is less effective for road area recognition with complex backgrounds. Aiming at the problem that trees block the road, a residual U-shaped network is proposed. It adds a residual module on the basis of the U-shaped network, and increases the network depth without affecting the image resolution. However, this network structure only solves the small-scale occlusion problem. In order to deal with small sample learning for occlusion regions, Zhang et al. [17] presents a road extraction method from aerial images using a generative adversarial network. The issue lies in the unstable learning in the adversarial process. In terms of the generalization ability, Senthilnath et al. [18] provide the conditional generative adversarial network followed by an ensemble classifier. Their work achieves the transfer learning ability between different unmanned aerial vehicle (UAV) remote sensing data. However, the road condition in UAV images is present in relatively single geometric shapes with fewer curves. To increase the scale and types of roads, Cira et al. [19] design a deep learning-based framework for road extraction at the scale of the national territory. As mentioned by [19], their accuracy decreases in urban and hilly areas caused by the increase presence of occlusions.

Researchers have tried to combine traditional methods with deep learning methods, for example, combining deep learning with spatial correlation to improve the accuracy of road extraction in urban contexts. In addition, thinning methods, geometric analysis, and level sets are also integrated into the post-processing stage of deep learning for improving the extraction accuracy. Compared with traditional methods, deep learning methods have the advantages of high automation and strong generalization, but deep learning methods require a large number of high-precision data sets. In addition, deep learning training takes a long time, and the road extraction convergence is still a challenge.

**Fig 1:**
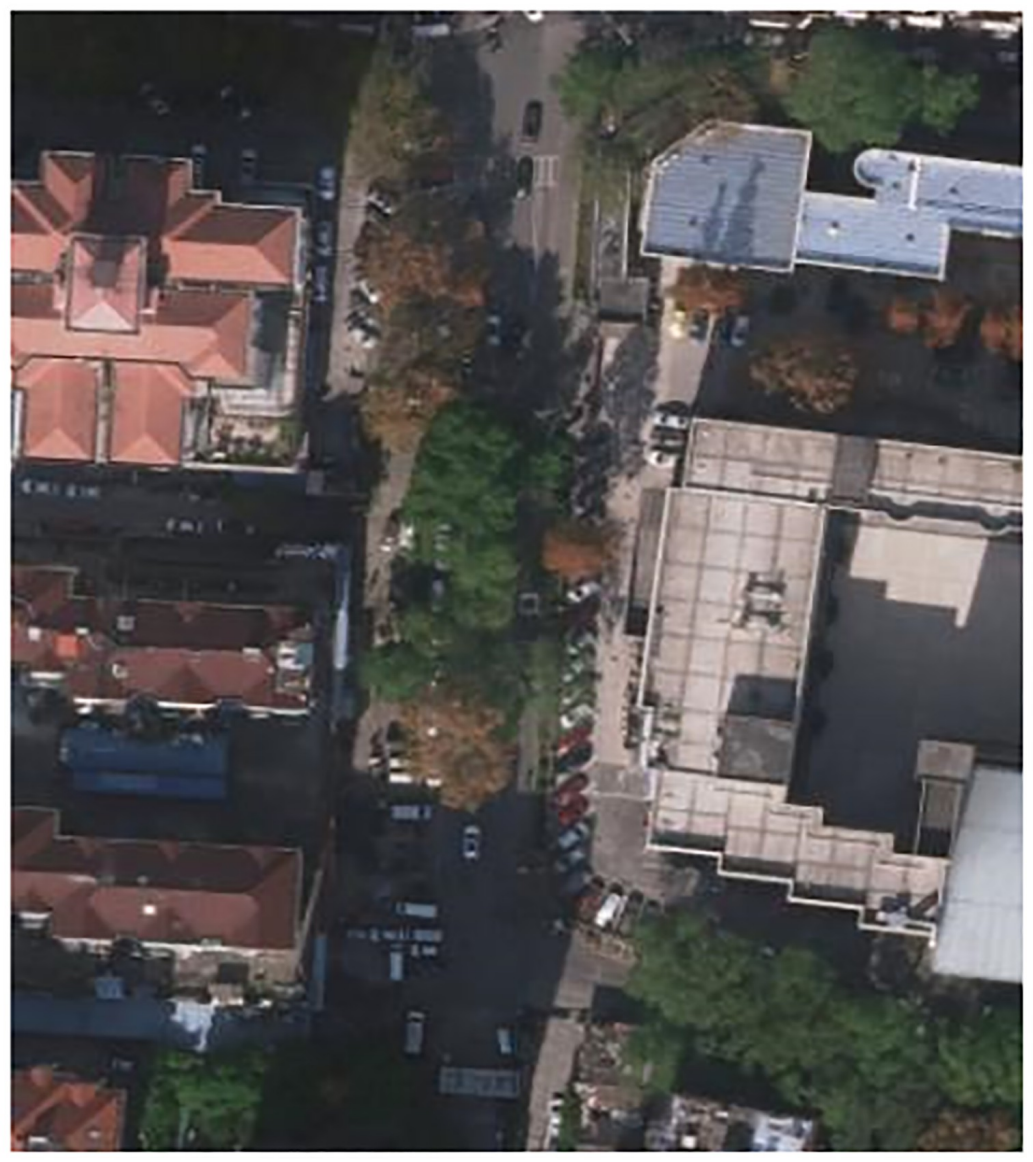
Road regions with occlusion problems.

Compared with rural roads, urban road environment is more complex and dense. There are more lanes on urban roads, such as vehicles, indicator lines, and tall buildings along the road. Therefore, the challenges of high-resolution remote sensing images for road extraction is the occlusion problem as shown in Fig 1. Buildings and trees around the road or their shadows will cause false edges or intermittent edges, when the road image is used for edge detection. This will cause the extraction results to be discontinuous and incomplete, because the similarity of many ground objects present a linear feature, such as rivers and city walls. This causes misclassification of extraction results and the accuracy is not high. The types of road surfaces, lengths, widths, and curvatures of roads will all be different, which makes the gray levels of different roads not similar, causing certain difficulties for road extraction. In addition, the limitations of the deep learning model itself also have a certain impact on the road extraction effect. The remote sensing image data set has a small amount of data and a small scale, which makes the model training difficult.

## The proposed road extraction algorithm

### The optimized pix2pix model

In order to provide images with more road features, the first step is to design a new model for the following detection step. Compared with classical image conversion methods, we uses adversarial training for training and optimization. That is, in a framework containing two neural networks, a generator (Generator) is responsible for converting the input image into a target image, while the other discriminator (Discriminator) is responsible for evaluating the output of the generator. In conditional GAN networks [20], the loss function is usually formulate as

$$\begin{array}{c} L_{0}\left(G,D\right)=E_{x,y}\left[\text{log}\;D\left(x,y\right)\right]+E_{x,z}\left[\text{log}\;\left(1-D\left(x,G\left(x,z\right)\right)\right)\right] \end{array}$$ (1)

where *G* is the generator and *D* is the discriminator. In order to reduce blurring and occlusion caused by trees, we add the *L*_1_ and *L*_2_ distance in the loss error evaluation as

$$\begin{array}{c} L_{1}\left(G,D\right)=E_{x,y,z}[||y-{G\left(x,z\right)||}_{1}+||y-{G\left(x,z\right)||}_{2}] \end{array}$$ (2)

The final object function is

$$\begin{array}{c} G=\text{arg}\;\underset{G}{\text{min}\;}\underset{D}{\text{max}\;}(L_{0}\left(G,D\right)+\lambda \times L_{1}\left(G\right)) \end{array}$$ (3)

where λ is the coefficient between two loss functions, which is set as 0.8 in this study.

Our road extraction is mainly through image semantic segmentation by the convolutional neural network (CNN), which divides the input image into multiple semantic regions. In road extraction, CNN can identify and separate the road area in the image, so as to realize road detection and extraction. Both the generator and the discriminator used in this work adopt the convolutional neural network (CNN) structure. The generator consists of several convolutional layers and deconvolutional layers, where convolutional layers are used to extract low-level features, and deconvolutional layers are used to restore high-level features. The discriminator uses operations such as convolutional layers and pooling layers to classify the image output by the generator to determine whether it is similar to the real target image. Hence, our network consists of two parts: a generator model and a discriminator model. We produce reasonably output through mutual game learning between two modules.

It should be noted that both the generator and discriminator are trained during the training process, so that the generator can learn better feature representations and produce more realistic road maps. During the training process of the model, its generator and discriminator continuously optimize themselves through mutual game. The core of the optimized pix2pix model is the generator and discriminator. This experimental study defines the generator as consisting of 6 downsampling, 5 upsampling, and 1 output layer. The optimized pix2pix model is actually similar to GAN, which changes the output of the discriminator of the general GAN. Its final output is a matrix, where each block represents the probability of a patch. The proposed pix2pix model has a powerful image conversion function, the image conversion is to translate the image as shown the following experiments.

### The fusion model

Compared with the traditional convolutional neural network (CNN), we adopt a new network structure, which is a combination of “Dense connection” and “Residual connection”. In this way, the accuracy and stability of the model can be effectively improved, and the phenomenon of over-fitting will be reduced. Specifically, the proposed fusion model consists of two parts: encoder and decoder. The encoder adopts a densely connected network (DenseNet) [21] structure, which contains 4 dense blocks (Dense Block), each of which consists of several convolutional layers and pooling layers. Within each dense block, the output of previous convolutional layers is concatenated with the input of subsequent convolutional layers to increase feature interaction and sharing. The decoder uses the deconvolution layer and advanced operations to gradually restore the feature map. The feature map is extracted by the encoder to the size of the original image, thereby realizing the division of image levels.

**Fig 2:**
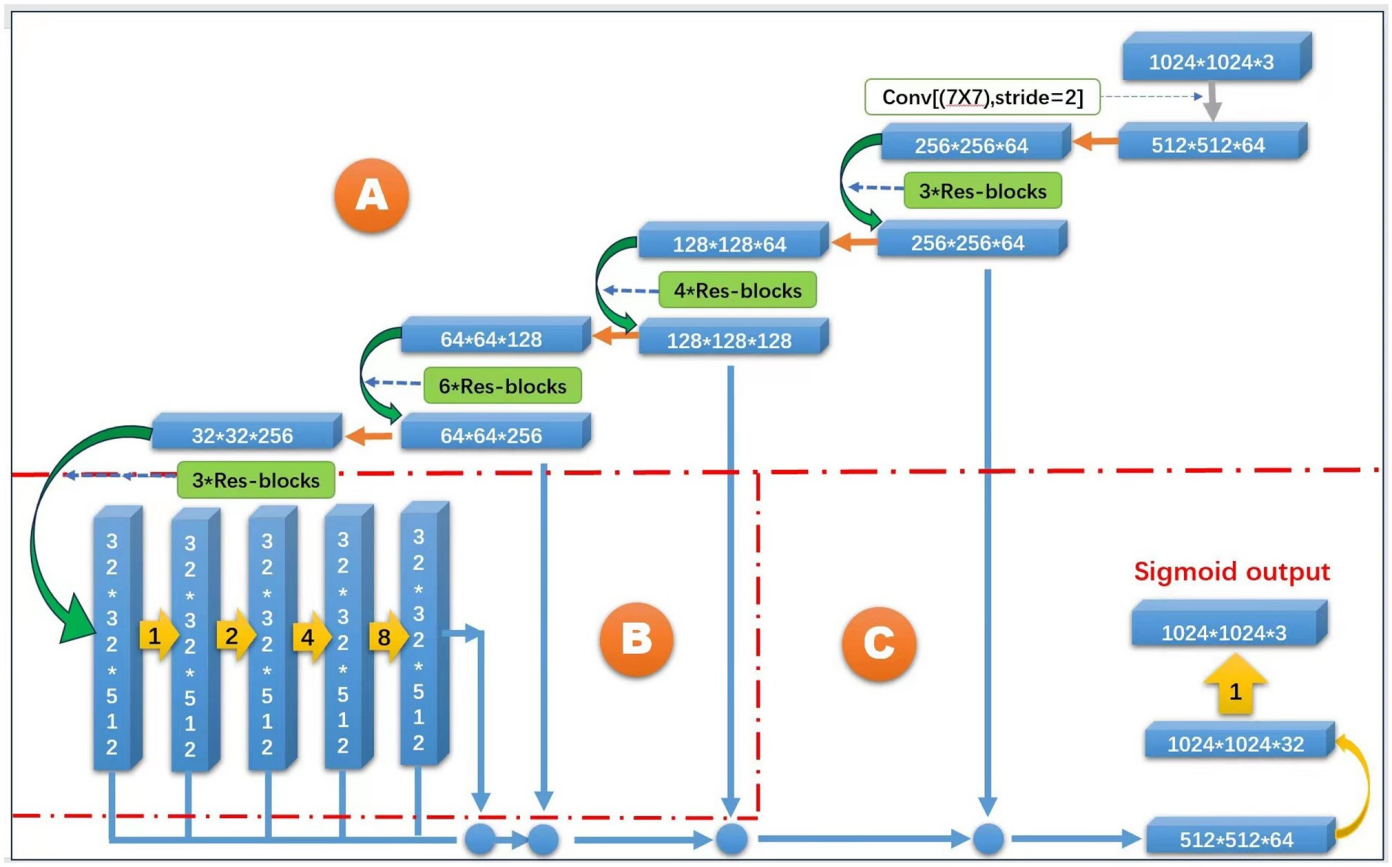
The proposed network structure.

In addition, we also use a combination of residual connection (ResNet) [22] and dense connection to ensure the stability and accuracy of the model. Specifically, a short-circuit connection (Short Connection) is added between the input and output of each dense block, so that the feature map of the previous layer is directly passed to the feature map of the later layer as shown in Fig 2, which promotes the transfer and sharing of information. The original image is taken as an input in the proposed model and converted into a vector in the latent space by an encoder. Subsequently, the decoder converts this vector back to a road map, outputting a result similar to a real road map. The core of the proposed model is the generator and discriminator. The generator maps the input image to the output image, and the discriminator is responsible for evaluating the difference between the generator’s output and the real image. In terms of road information extraction, the proposed model can convert the input original image into a road map, and the specific implementation process is as follows.

As shown in Fig 2, we have three different modules in the network. Module A based on a common ResNet to provide a stable learning stage in the training, i.e. avoid gradient vanished in the optimization. In order to fuse more features in the learning, we add two more modules B and C in the network, which directly add features to the following stages. Hence, feature map information will be kept in the learning. Actually, one can repeat our structure to obtain even more deep networks. However, our experiments indicate that the accuracy is not exactly proportional to the number of layers.

### The proposed two-step road extraction

First, take the original image as input for the optimized pix2pix model, and convert it into vector in potential space through encoder. Subsequently, the decoder converts this vector back into a road map and outputs a result similar to a real road map. It should be noted that the generator and discriminator are trained at the same time during the training process, so that the generator can learn better feature representation and produce more realistic road maps. During the training process of the model, its generator and discriminator continuously optimize themselves through mutual game.

We perform well when the data is sufficient, because the proposed pix2pix model can learn complex features and patterns from a large amount of image data through confrontational training. At the same time, the model is trained by the confrontation process between the generator and the discriminator, which can effectively reduce the interference of noise, illumination, occlusion and other factors on the extraction of road information. The image output by the optimized pix2pix model is very similar to the real target image, which can intuitively reflect information such as the location, shape, and size of the road. Therefore, the optimized pix2pix model has a great advantage in the interpretability of road information extraction.

It is worth noting that there are two special branches in the proposed network: a backbone network and an up-sampling network. The backbone network uses a pre-trained model such as ResNet or VGGNet to extract features of the input image through multiple convolution layers and pooling layers. These features can abstractly represent different image elements, such as edges, corners, lines, and so on. The final output of the backbone network will be used as the input of the up-sampling network. The up-sampling network is the opposite of the backbone network, which expands the low-resolution feature map to the size of the original image. We introduce the SkipConnection mechanism and the up-sampling network to multiple convolutional layers and deconvolutional layers, so that the network can simultaneously exploit different levels of feature information. Another important design is the prediction module, which use feature maps of different resolutions for information fusion to extract more accurate information. Specifically, the prediction module concatenates the feature maps of three different resolutions in the backbone network and the feature maps of two different resolutions in the up-sampling network.

## Experiment and analysis

### Datasets description

It should be noted that the generator and the discriminator are trained simultaneously during the training process so that the generator can learn better feature representations and produce more realistic road maps. During the training process, the process of its generator and discriminator continuously optimizing themselves. The test coding environments are shown in Table 1.

**Table 1:** Experimental environments.

OS	CUDA	pytorch	Python	cudatoolkit	torchaudio	torchvision
Windows11	8.0	1.8.1	3.8.16	10.2.89	0.7.0	0.8.0

The experiment hardware is carried out on the Windows 11 operating system, with a memory size of 64G. We also utilize a GPU powered by an NVIDIA GeForce 3060.

In terms of data filtering, in order to remove noise, shadows, clouds and other interference factors in the image and ensure image quality, a method based on ground control points (GCP) is used for registration and correction. In terms of data correction, we use image fusion technology, i.e. band weighted average. Multiple images are merged into a complete image. Projection transformation and other operations are jointly used to ensure the geometric accuracy of the image and solve the problem of edge inconsistency.

During the training process, we adopted a combination of adversarial Loss function and L_1_ Loss function. The adversarial loss function is used to measure the discriminator’s evaluation of the output of the generator, and the L_1_ loss function is used to measure the distance between the output of the generator and the real target image. Through the joint action of these two loss functions, the learning and optimization of the model can be facilitated, and the quality and accuracy of image conversion can be improved.

**Fig 3:**
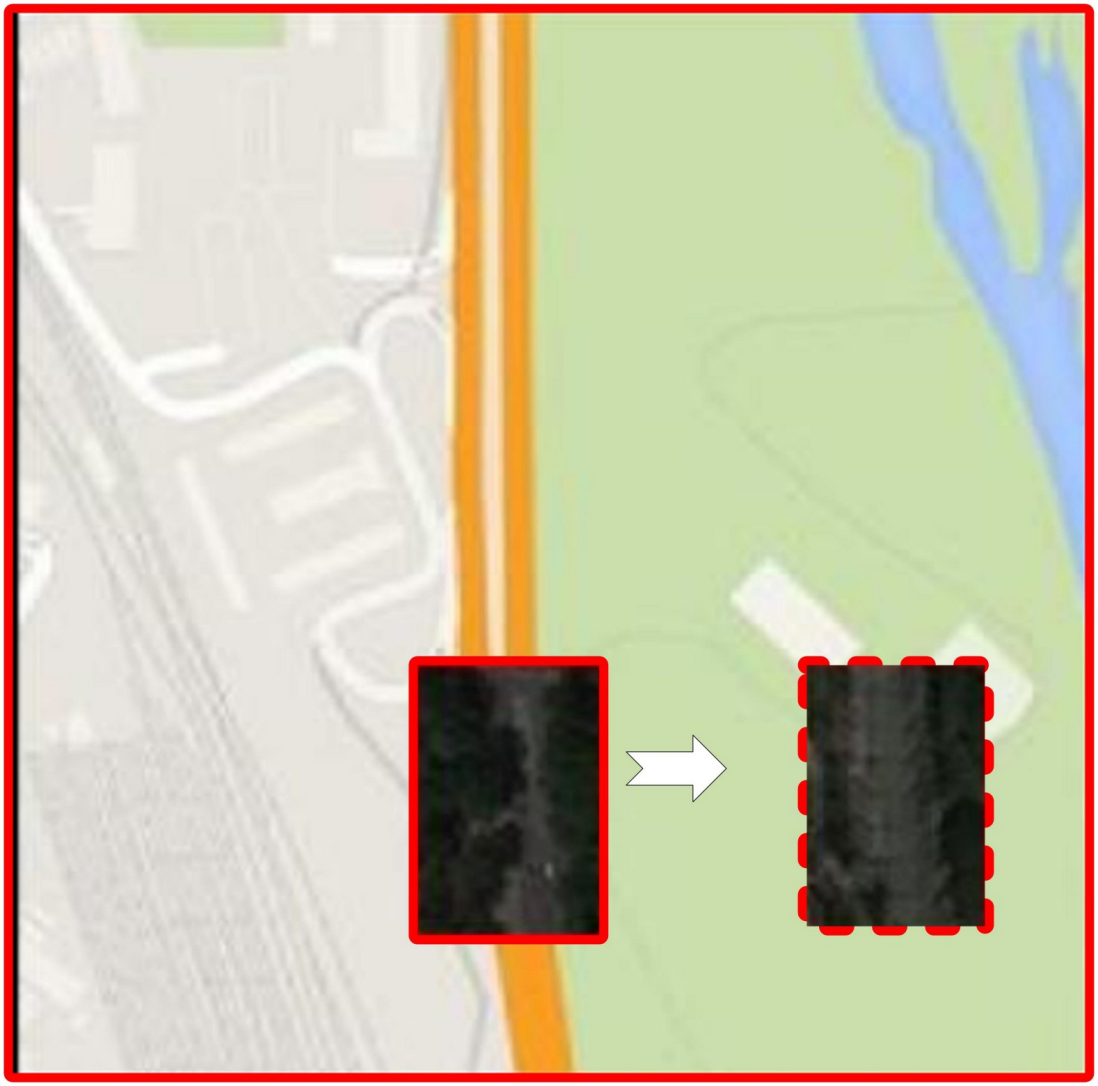
Road map (left), the output features of the optimized pix2pix (middle) and the output features of the classical pix2pix (right) at the beginning of training(Created by Microsoft power point).

At the same time, the optimized pix2pix model based on the GAN architecture performs well when the road is partially occluded. It is not difficult to see from Fig 3 that the image can remove trees that block the road in the early stage of training. But for the classical pix2pix model, its main function still focuses on the translation of pictures, so the clarity of the pictures needs to be improved.

In the analysis, the first data set comes from the DeepGlobe-Road-Extraction-Challenge data set (http://deepglobe.org/resources.html) [23]. The DeepGlobe data set is a remote sensing image jointly released by Washington University, Stanford University, and the University of Montreal. The data set covers high-resolution satellite imagery worldwide and includes 3 tasks: road extraction, building detection, and land surface cover classification. For different tasks, the DeepGlobe dataset provides corresponding annotation information to facilitate the training and testing of deep learning models. This research uses the road to extract the track, the image size is 1024 × 1024, and the three-channel format of PNG and JPG is used for representation. It contains 1200 test images and 5000 training images. The data set is large in scale and can meet the needs of many large-scale remote sensing image processing tasks. In DeepGlobe dataset, the Train set contains SAT and MASK, we use the Train set as the initial dataset and divides it into Train and Valid datasets for training and testing, respectively. Due to limitations in computer hardware performance and training time, the initial epoch_Time is about one hour, the result is not satisfactory and it is difficult to meet the experimental requirements. Subsequently, we use non-overlapping image cropping strategy to segment the image, and without affecting the overall data volume, the data volume increased to several times the previous one. The epoch_Time was adjusted to 20 minutes. Besides, in order to avoid the risk of overfitting to the training set, this experiment enhances the experimental data through image flipping, vertical flipping, diagonal flipping, color jitter, image shift, scaling, and other methods. Our experiment uses batchsize = 4. Our model has certain advantages in handling attributes such as narrowness, connectivity, complexity, and long span of roads to a certain extent while maintaining detailed information. It presents in high-quality for roads with clear boundaries. There may even be situations where the label is not marked but the recognition is correct.

The key experimental parameters in the learning stage is shown in Table 2.

**Table 2:** Experimental parameters in the learning stage.

Image	BatchSize	Epoch	Time (mins)	Learning Ratio	Optimizer
1024 × 1024	4	220	20	0.0001	Adam

The proposed method has advantages in handling the narrow, connectivity and complexity roads while maintaining detailed information. For roads with clear boundaries, there is a well extraction effect, and even cases where the label is not marked but the recognition is correct. However, we still has road connection issues. For road images with blurred boundaries, its extraction effect is low. For images with building shadows, there are shortcomings in distinguishing small paths and building shadows.

The second data set is the Massachusetts Road Dataset (https://www.kaggle.com/datasets/balraj98/massachusetts-roads-dataset) [24]. The publicly available Massachusetts road data set consists of 1171 sheets with a resolution of 1m. This data set covers urban, suburban, and rural areas, with a total area of over 2600 square kilometers. We also convert images to 256 × 256 size. Then, 16800 images were obtained as the data set and divided into the validation set and training set at a ratio of 2:8. Our extraction results of the main road are clear and coherent. The overall road situation in the image is relatively clear, with significant differences in road and non road information. However, there is a problem of house shadow occlusion in the lower left and upper right corners of the original image, resulting in discontinuous road extraction results.

In the experiments of the two data sets, the pix2pix model and the fusion LinkNet model can achieve better performance in the road extraction task. Road information extraction from high-resolution remote sensing images is a complex task, which involves many aspects such as data preprocessing, feature extraction, model design and evaluation. Choosing an appropriate model is an important part of this, but it is not the only concern of the research. In the research, it is also necessary to pay attention to the selection and construction of data sets, the determination of evaluation indicators, and the control of experimental conditions to ensure the scientifically and credibility of the research. Therefore, the research on road information extraction should be problem-oriented, focusing on solving specific problems and seeking the effect of practical application, rather than just pursuing the performance of the model itself.

### Quantitative analysis

IOU (Intersection over Union) is an indicator used to evaluate the accuracy of image segmentation results, also known as the Jaccard coefficient [25]. The IOU index measures the ratio between the intersection and union of the predicted segmentation area and the real segmentation area. It is also one of the commonly used indicators for evaluating the performance of image segmentation models.

This experiment uses three indicators of 0_IOU, 1_IOU and MIOU to measure the performance of the model. 0_IOU, also known as the global IOU, which represents the average IOU of all pixels, and it is an indicator for globally measuring the accuracy of the segmentation model. Generally, the higher the 0_IOU, the better the performance of the segmentation model. 1_IOU, also known as the per-class IOU, which represents the IOU value for each class and is a metric for class-specific evaluation of segmentation models. The 1_IOU metric is very useful when more fine-grained evaluation is required for certain categories. MIOU, also known as the average IOU, which represents the average IOU of all categories and is an indicator for evaluating the overall accuracy of the segmentation model. Generally, the higher the MIOU, the better the overall performance of the segmentation model. Our quantitative evaluation results are shown in Table 3.

**Table 3:** Quantitative evaluation of results on DeepGlobe and Massachusetts dataset.

Dataset	ACC	1_IOU	MIOU	0_IOU
DeepGlobe	0.94	0.65	0.80	0.94
Massachusetts	0.96	0.74	0.85	0.95

As shown in Table 4, the original model L_0_ is prone to missing roads for roads with shadows, narrow roads, roads with small spectral differences, and roads blocked by obstacles. The above problems have addressed when we choose L_0_+L_1_ in the loss function.

**Table 4:** Ablation tests of different loss functions.

Loss	ImageSize	BatchSize	Epoch	MIOU	Peak MIOU	Standard deviation
L_0_	256 × 256	4	180	0.82	0.83	0.006
L_1_	256 × 256	4	150	0.65	0.72	0.01
L_0_+L_1_	256 × 256	4	250	0.86	0.91	0.005

It should be clarified that the optimized pix2pix model can learn complex features and patterns from a large amount of image data through adversarial training. At the same time, the model is trained using the adversarial process between the generator and discriminator, which can effectively reduce the interference of noise, lighting, and occlusion factors on road information extraction. The stability demonstrated in this experiment is strong. The image output by the pix2pix model can intuitively reflect information such as the position, shape, and size of the road. Therefore, the proposed pix2pix model has significant advantages in terms of interpretability in road information extraction.

### Robustness test in real scenes

**Fig 4:**
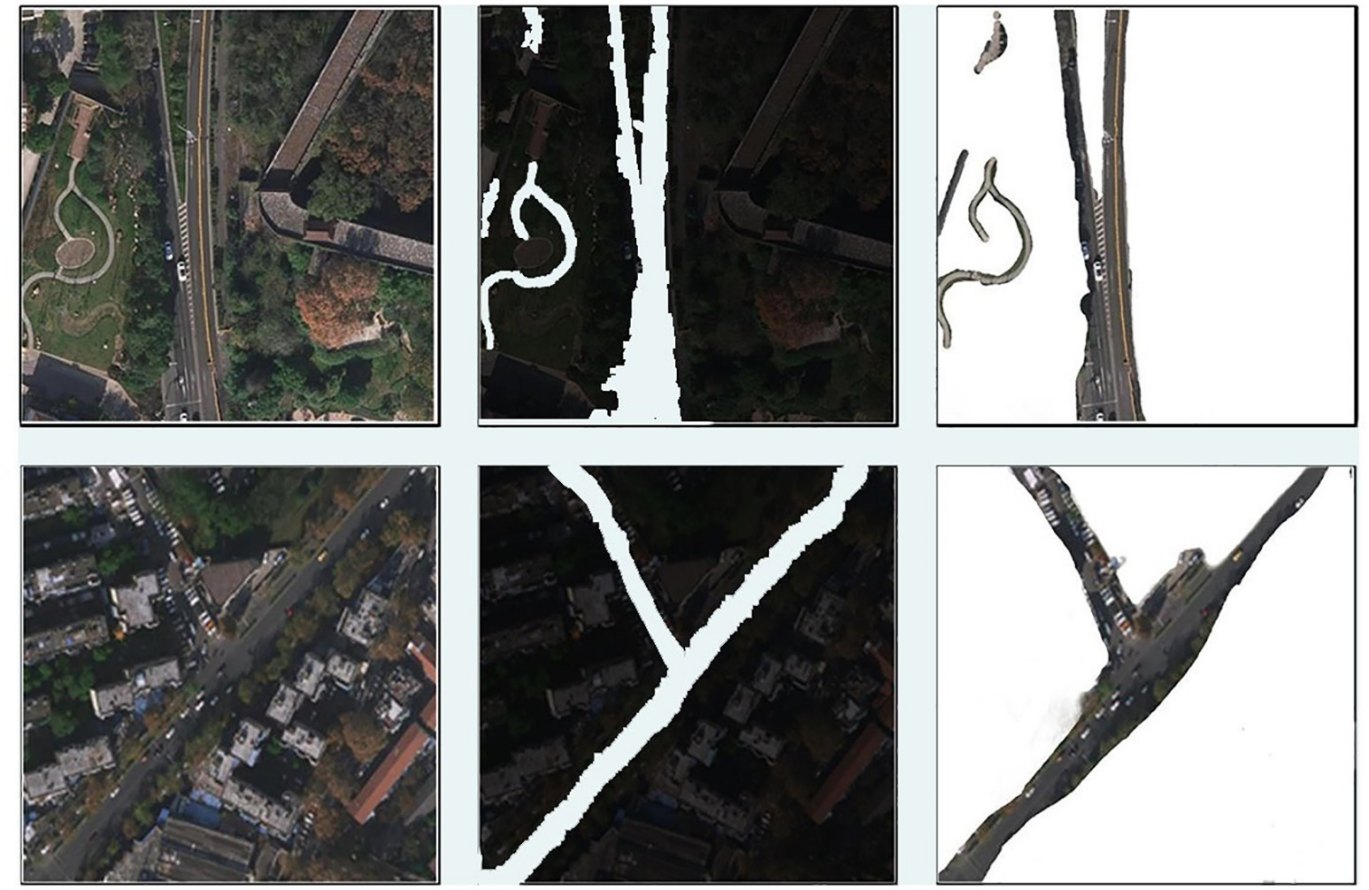
Display of road extraction results in our data set. The first column is the collected remotes sensing, the second column is the extraction results and the third is the road images based on the second column by using morphological processing.

In practical, we collect the following road data set, which has a high resolution, a large amount of ground feature information. There are many lanes of the road itself, public transport and other indicator lines and vehicles on the road. Fig 4 shows the road extraction results in a road scenario. For the extraction of the main road section, except for the indicator line section, the results are relatively continuous. The roads in this data set area themselves belong to the urban road network scenario. Even if the main road is wide, the extraction of the road with vehicle occlusion is discontinuous.

Fig 4 displays road extraction results in our data set. The first column is the collected remotes sensing, the second column is the extraction results and the third is the road images based on the second column by using morphological processing. Table 5 shows the detected correct roads in the different regions, including main road, branch road and path.

**Table 5:** Regional classification results.

NO.	Scene	Number	Correct	Ratio	Error
1	Main roads	55	52	94%	17
2	Branch roads	43	31	72%	7
3	Path roads	23	16	70%	20

As shown in the Table 5, it can be understood that the results of region classification are not the same for different scenarios. In main roads, there is a relatively large proportion of the road, and most of them are two-way four lane main roads. The recognition accuracy is high, and most of them can cover the edge parts of the road. In the images of the branch roads, there are fewer main lanes, and the roads between the residential areas are mostly double lanes. There are large areas of trees covering the roadside, which is difficult for the human eye to detect and the recognition accuracy is low. In paths, roads cover a relatively small area, and there are also forest roads that are not easily visible. The path roads are relatively open and exists in areas with very similar roads, which can easily lead to the identification of non-road areas as road areas.

## Conclusion

This paper mainly elaborates on the research and application road extraction in remote sensing images. We present two relevant data sets in the analysis of model training and prediction results. According to the results, the advantages and disadvantages of the extraction methods were summarized, and ablation tests were conducted. Our innovation of this study lies in:

1. Breaking away from the limitations of traditional road information extraction, the optimized pix2pix model network is used for image translation to obtain the required images. The pix2pix model outputs images that are very similar to the real target image, rather than traditional black and white road images. It can intuitively reflect the location, shape, and size of the road. Higher practicality and applicable value.
2. A semantic segmentation model, fusion LinkNet, was present to optimize the road extraction at a low time-consuming and well convergence.

Future works focus on the following 2 points. First, multiple algorithms or optimization training methods can be further tried to adjust hyper parameters to improve the training upper limit and over-fitting problem. Second, the proposed fusion LinkNet still has recognition errors and road connection issues, and the number of feature extraction layers is not high. For example, the model may be affected by changes in lighting or unclear road textures, resulting in inaccurate model predictions. At the same time, the model also requires a large amount of computing resources and time to train, and it does not perform well in this experimental machine. The training time is too long, and fine adjustments to the model parameters are required to achieve good performance.
